# *GenomeLandscaper*: Landscape analysis of genome-fingerprints maps assessing chromosome architecture

**DOI:** 10.1038/s41598-018-19366-2

**Published:** 2018-01-18

**Authors:** Hannan Ai, Yuncan Ai, Fanmei Meng

**Affiliations:** 1 State Key Laboratory for Biocontrol, School of Life Sciences, Sun Yat-sen University, Guangzhou, Guangdong, 510275 China; 2 Department of Electrical and Computer Engineering, College of Engineering, University of Illinois at Urbana-Champaign, Urbana, IL 61801 USA

## Abstract

Assessing correctness of an assembled chromosome architecture is a central challenge. We create a geometric analysis method (called *GenomeLandscaper*) to conduct landscape analysis of genome-fingerprints maps (GFM), trace large-scale repetitive regions, and assess their impacts on the global architectures of assembled chromosomes. We develop an alignment-free method for phylogenetics analysis. The human Y chromosomes (GRCh.chrY, HuRef.chrY and YH.chrY) are analysed as a proof-of-concept study. We construct a galaxy of genome-fingerprints maps (GGFM) for them, and a landscape compatibility among relatives is observed. But a long sharp straight line on the GGFM breaks such a landscape compatibility, distinguishing GRCh38p1.chrY (and throughout GRCh38p7.chrY) from GRCh37p13.chrY, HuRef.chrY and YH.chrY. We delete a 1.30-Mbp target segment to rescue the landscape compatibility, matching the antecedent GRCh37p13.chrY. We re-locate it into the modelled centromeric and pericentromeric region of GRCh38p10.chrY, matching a gap placeholder of GRCh37p13.chrY. We decompose it into sub-constituents (such as BACs, interspersed repeats, and tandem repeats) and trace their homologues by phylogenetics analysis. We elucidate that most examined tandem repeats are of reasonable quality, but the BAC-sized repeats, 173U1020C (176.46 Kbp) and 5U41068C (205.34 Kbp), are likely over-repeated. These results offer unique insights into the centromeric and pericentromeric regions of the human Y chromosomes.

## Introduction

Centromeres and telomeres of mammalian genomes are yet-untouchable large-scale repetitive regions due to technical constraints of sequencing and assembling^1–3^, despite that straightforward assembling was improved by *de novo* assemblers like Celera^4–6^, SOAPdenovo^7^, Supernova^8^, Canu^9^, HINGE^10^ and Recon^11^ equipped on platforms such as Sanger, Illumina, PacBio and Oxford Nanopore. It is challenging to retrospectively assess the correctness of an assembled chromosome architecture because no “true” sequence can be referred to^1–3^ as well as the data-driven analysis is hampered by a computing burden of base-to-base alignment at a large scale. Without a “true” reference, the reference-alignment based assembling and adjusting are often inevitably trapped by a logic paradox: which one is correct if two queries are inconsistent with one another? are they both correct or incorrect if they are consistent? These make up a central challenge in the post era of the 1,000 genomes project^2,3^. Few methods were dedicated to retrospectively assess the global architectures of assembled chromosomes to date.

The human genomes have typical assemblies, such as GRCh from a mixture of diploids (Global)^12,13^, HuRef from an individual diploid (USA)^4–6^ and YH from an individual diploid (Asia)^7^. They are technical and biological representatives. Only GRCh has been constantly updated, standing for the human reference genome^12,13^, which is arguably the best assembled mammalian genome to date^1^. However, the centromeres and telomeres were not adequately addressed^14–16^ and masked by Ns as gap placeholders, as of the 13rd patch release of GRCh37^17,18^. Recently, centromere model representations were created by graph-based simulations^16^ and introduced in the current human reference GRCh38 assembly used for mapping target short-reads^1^. It opened a door to simulate the yet-untouchable centromeres of mammalian genomes^1,16^. Retrospectively assessing such modelled centromeres in the human reference genome becomes an urgent interest, which intrigues the field to create new methods.

This study addresses the above critical issues and aim to (1) display discrepancies among multiple assemblies of a chromosome, (2) detect misassembled segments of its chromosome architecture, (3) delete the misassembled segments, and (4) decompose the segment into sub-constituents and trace their homologues that contributed to the misassembling. As such, one can detect misassembling and determine to replace a misassembled artefact or maintain a true intrinsic segment. Our hypotheses are: (1) there should be a landscape compatibility among relatives under comparison, and (2) the more the relatives were compared at a large scale, the easier the misassembled architectures could be detected in a big-picture view, thus overcoming the aforementioned central challenge. Based on our *GenomeFingerprinter* algorithm^19^, here we establish a method (called *GenomeLandscaper*) to construct a galaxy of genome-fingerprints maps (GGFM), which comprises a set of genome-fingerprints maps (GFM) that are simultaneously constructed for a set of chromosomes under comparison. Hence we can compare a set of chromosomes in a big-picture view at a large scale. To compare a number of large and divergent genomes, we also develop an alignment-free method for phylogenetics analysis. As a proof-of-concept study, we create a GGFM for the human Y chromosomes (GRCh.chrY^12,13^, HuRef.chrY^4–6^ and YH.chrY^7^) and conduct assessments on their global architectures. This study establishes a method to retrospectively assess the correctness of assembled chromosome architectures by means of evaluating the quality of their multiple assemblies, which is crucial to assess, re-construct and use complex genomes.

## Results

### Principles of the *GenomeLandscaper* method

**Figure 1:**
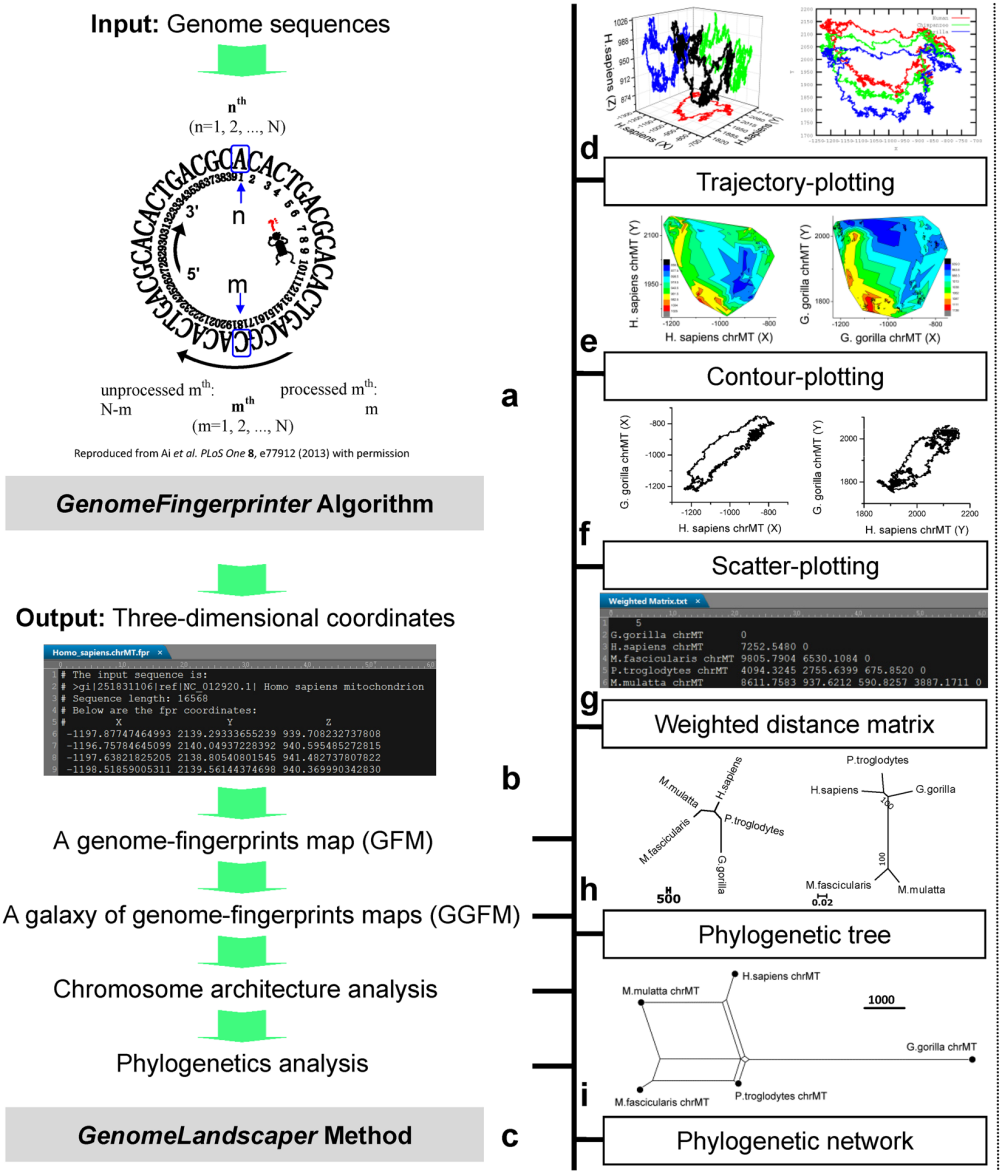
Overview of computational framework of the *GenomeLandscaper* method. The steps of working flow with key features are illustrated. Explanations are given in the main text.

To conduct landscape analysis of genome-fingerprints maps (GFM) and retrospectively assess the global architectures of assembled chromosomes, we establish the *GenomeLandscaper* method based on our *GenomeFingerprinter* algorithm^19^. The steps of working flow with key features are illustrated by using the primates mitochondria genomes (<17.0 Kbp, kilos of base pairs) including *Homo sapiens*, *Pan troglodytes*, *Gorilla gorilla*, *Macaca fascicularis* and *Macaca mulatta* (Fig. 1).

#### Feature 1. Calculation of three-dimensional coordinates

Our *GenomeFingerprinter* algorithm circularises a linear sequence (Fig. 1a) to avoid interference from arbitrary cut-off sites of the sequence^19^. It defines a set of functions for the three-dimensional coordinates (*Xn*,*Yn*, *Zn*) (Fig. 1b), where *n* is the order number of a base (A, T or U, G and C) of a sequence (in length of *N*)^19^:

1 $$\{\begin{array}{c}Xn=f(An+Gn)-f(Cn+Tn)\\ Yn=f(An+Cn)-f(Gn+Tn)\\ Zn=f(An+Tn)-f(Cn+Gn)\end{array}$$

Each component of the coordinates is a function of distribution difference between the assigned two base-types, reflecting the distribution bias between them. We speculate that a certain continuous distribution bias along with a given sequence should yield a line along an axis. A line along the X axis illustrates a continuous distribution bias of purine (A plus G) over pyridine (C plus T); a line along the Y axis indicates a continuous distribution bias of amino-nucleotides (A plus C) over keto-nucleotides(G plus T); and a line along the Z axis demonstrates a continuous distribution bias of weak hydrogen bonds (A plus T) over strong hydrogen bonds (C plus G).

#### Feature 2. Construction of a genome-fingerprints map (GFM)

Using the three-dimensional coordinates (Fig. 1b), we can transform a genome into a genome-fingerprints map (GFM) by trajectory-plotting (Fig. 1d), contour-plotting (Fig. 1e) and scatter-plotting (Fig. 1f), respectively.

#### Feature 3. Construction of a galaxy of genome-fingerprints maps (GGFM)

A set of GFMs can be simultaneously constructed for a batch of genomes under comparison in order to create a galaxy of genome-fingerprints maps (GGFM). Such graphic visualisations are expensive computations for large genomes.

#### Feature 4. Chromosome architecture analysis on the GGFM

The 3D-map (Fig. 1d) and the set of 2D-maps (Fig. 1d, e) can represent the given genome, respectively. We operate map-to-map comparison, instead of alignment-based base-to-base comparison. This alignment-free method relieves computation burdens and allows us to compare a number of large genomes and divergent genomes in a big-picture view at a large scale.

#### Feature 5. Calculation of a weighted distance matrix

The geometric centre ($\bar{X}$, $\bar{Y}$, $\overline{Z}$) of a GFM solely represents the given genome and is regarded as a point in three-dimensional space, thus a weighted Euclidean distance between two points, the *i* th ($\bar{X}i$, $\bar{Y}i$, $\overline{Z}i$) and the *j* th ($\bar{X}j$, $\bar{Y}j$, $\overline{Z}j$), is calculated by the formula:

2 $$\begin{array}{rcl}{d}_{(i,j)} & = & \sqrt{\sqrt{{\sigma }_{Xi}.{\sigma }_{Xj}}.{(\bar{X}i-\bar{X}j)}^{2}+\sqrt{{\sigma }_{Yi}.{\sigma }_{Yj}}.{(\bar{Y}i-\bar{Y}j)}^{2}+\sqrt{{\sigma }_{Zi}.{\sigma }_{Zj}}.{(\overline{Z}i-\overline{Z}j)}^{2}}\\ i,j & = & 1,2,\mathrm{...},M\end{array}$$

where *σ*_*X*_, *σ*_*Y*_ and *σ*_*Z*_ is the standard deviation for each axis; and *M* is the number of genomes to be compared, which determines a *M* × *M* symmetric matrix whose elements are *d*_(*i,j*)_. We weight the geometrical mean of radiuses of the circularised 3D-maps (Fig. 1d) to discriminate a pair of overlapped 3D-maps (sharing a geometric centre with different shapes).

To consider the effect of angles on the eigenvectors (*C*_*k*_^*i*^, *C*_*k*_^*j*^) between two genomes, we integrate other factors into the weighted Euclidean distance matrix, referring to^20^:

3 $$\cos \,{\theta }_{(i,j)}^{k}=\frac{{C}_{k}^{i}\cdot {C}_{k}^{j}}{|{C}_{k}^{i}|\cdot |{C}_{k}^{j}|},i,j=1,\,2\,,\mathrm{...},\,M;k=X,Y,Z$$

4 $${\theta }_{(i,j)}^{k}=\arccos (\cos \,{\theta }_{(i,j)}^{k}),i,j=1,2,\mathrm{...},M;k=X,Y,Z$$

5 $${{\rm{\Theta }}}_{(i,j)}={\theta }_{(i,j)}^{X}+{\theta }_{(i,j)}^{Y}+{\theta }_{(i,j)}^{Z},i,j=1,2,\mathrm{...},M$$

6 $${D}_{(i,j)}={d}_{(i,j)}.{{\rm{\Theta }}}_{(i,j)},i,j=1,2,\mathrm{...},M$$

Hence a final *M* × *M* symmetric matrix, whose elements are *D*_(*i,j*)_, is created (Fig. 1g).

#### Feature 6. Construction of a phylogenetic tree and a phylogentic network

The weighted distance matrix (Fig. 1g) can be transformed into a phylogenetic tree (Fig. 1h) and a phylogenetic network (Fig. 1i) by conventional software^21–25^. We construct a phylogenetic tree (Fig. 1h, left) using FastME software^21^ based on our weighted distance matrix. We construct a bootstrap consensus tree (Fig. 1h, right) using the MEGA6 package^22^ under the minimum-evolution (ME) model. Two un-rooted trees are approximate to one another, indicating that our alignment-free and bootstrap-free method has an adequate approximation (Fig. 1h). Such an approximation has an advantage in analysing a number of large genomes (e.g., Mbp, millions of base pairs) and divergent sequences (e.g., variations in size, gap, and divergence). If necessary, our method can be applied to analyse each set of disturbed sequences that are created by traditional bootstrap approaches^21–25^; but the traditional bootstrap approaches may not work when disturbing large genomes and divergent sequences due to algorithmic and computational constraints.

### Frameworks of using the *GenomeLandscaper* method

The frameworks of using the *GenomeLandscaper* method are exemplified by a proof-of-concept study on the human Y chromosomes (GRCh.chrY^12,13^, HuRef.chrY^4–6^ and YH.chrY^7^). We develop an itinerary throughout the next sections: (1) constructing a galaxy of genome-fingerprints maps, (2) detecting discrepancies among multiple assemblies, (3) deleting the misassembled chromosome architecture, (4) re-locating the deleted target segment, (5) tracing BACs of the deleted target segment, (6) tracing interspersed repeats of the deleted target segment, and (7) tracing tandem repeats of the deleted target segment. Notably, instead of intending to conduct the straightforward *de novo* assembling, our goal is to provide a novel method to retrospectively assess the correctness of assembled chromosome architectures by means of evaluating the quality of their multiple assemblies, so that one can detect misassembling and determine to replace a misassembled segment or maintain a true intrinsic segment.

### Constructing a galaxy of genome-fingerprints maps

**Figure 2:**
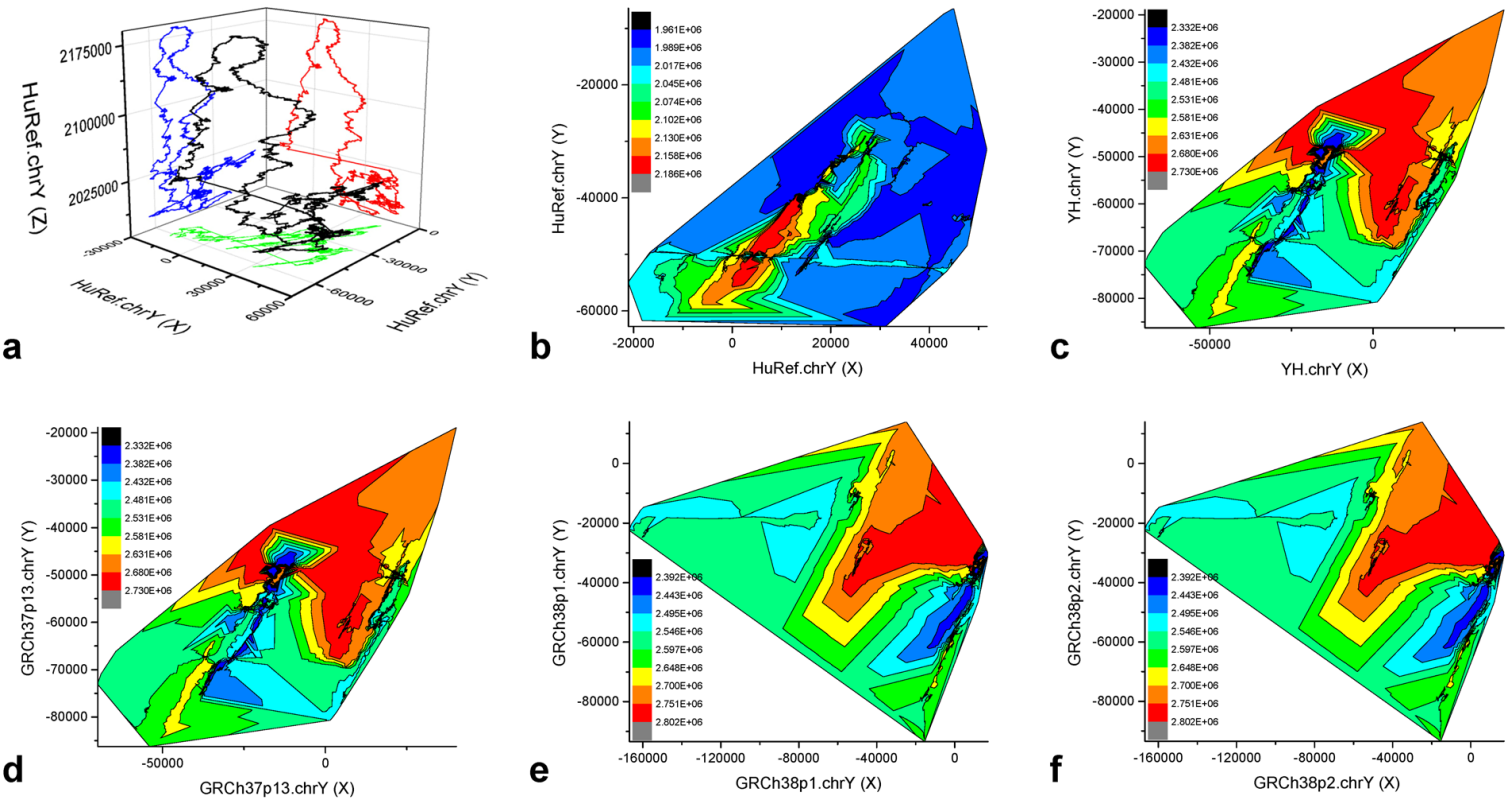
A galaxy of genome-fingerprints maps of the human Y chromosomes. A compact GGFM of HuRef.chrY (**a**) consists of one 3D-trajectory map (X–Y–Z, black) and three 2D-trajectory maps: (X–Y, green), (X–Z, red) and (Y–Z, blue). The 2D-contour (X–Y) maps (**b**,**c**,**d**,**e**,**f**) demonstrate high-resolution genome fingerprints. The colour-scales indicate variations of Z values (**b**,**c**,**d**,**e**,**f**). HuRef.chrY (**b**) is distinct. YH.chrY (**c**) and GRCh37p1.chrY (**d**) have no detectable difference. GRCh38p1.chrY (**e**), GRCh38p2.chrY (**f**) are identical. But GRCh37p1.chrY (**d**) is distinct from its two descendents (**e**,**f**).

A compact GGFM contains one 3D-trajectory map (X–Y–Z) and three 2D-trajectory maps (X–Y, X–Z, Y–Z) for at least one genome (Fig. 2a). Alternatively, we create a 2D-contour map (X–Y) (Fig. 2b–f) for each genome to demonstrate its high-resolution genome fingerprints. HuRef.chrY (18.18 Mbp) produces 0.92 GB (gigabytes) of coordinates. We took 72 hours (Fig. 2a) and 2 hours (Fig. 2b) to construct two figures for one genome HuRef.chrY (Fig. 2a,b), respectively, on a high performance workstation (Dell Precision T7600) with 192 GB (Gigabytes) physical memory installed with Origin Pro 9.0 (64-bit) software. Such two forms (Fig. 2a,b) are expensive to construct, which hampers their practical applications to a set of genomes under comparison.

**Figure 3:**
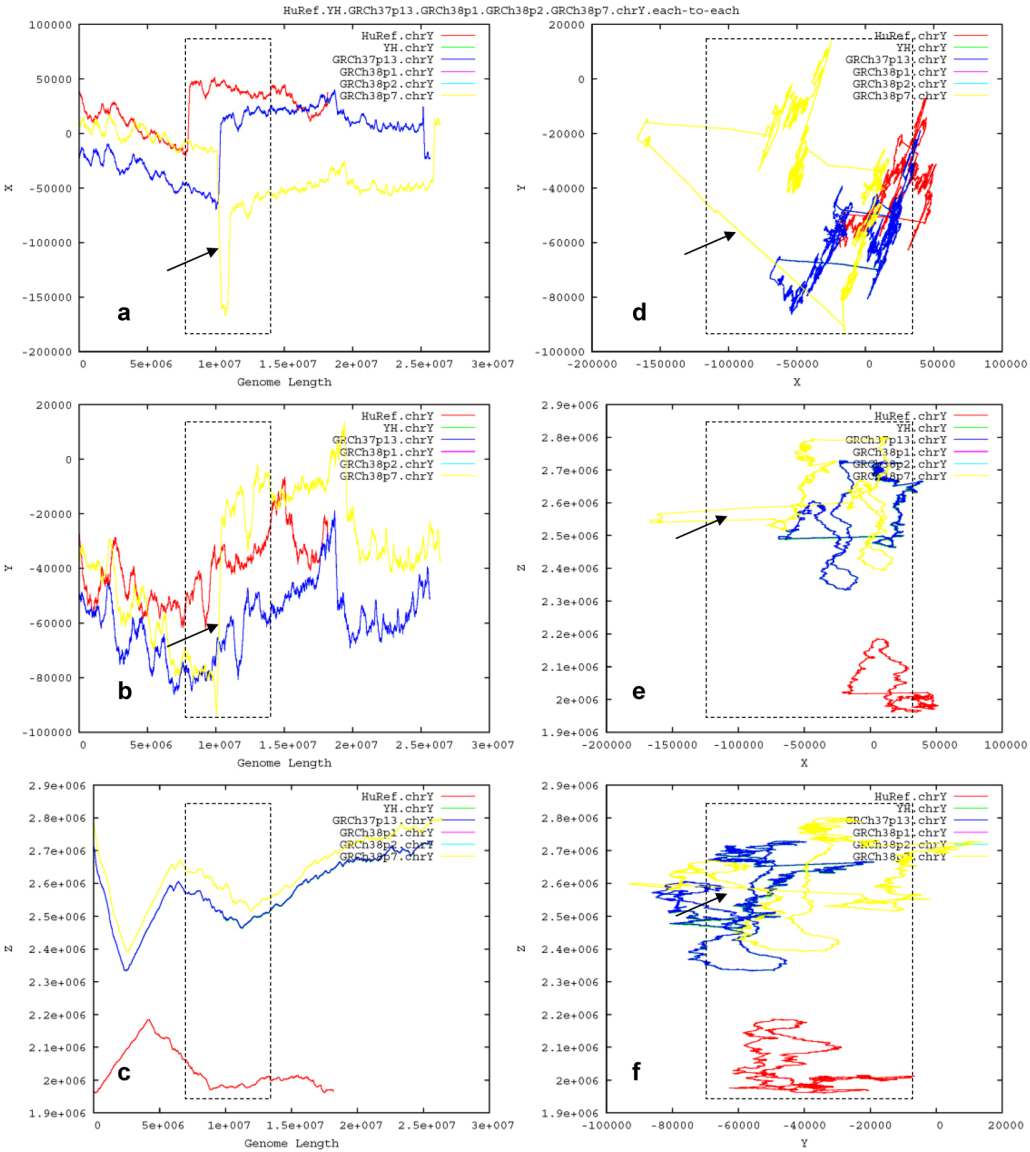
Comparison of global architecture among the human Y chromosomes. An alternative form of GGFM is presented. HuRef.chrY (red) is distinct due to its incompleteness of q-arm terminus. YH.chrY (green) is almost overlapped by GRCh37p13.chrY (blue). GRCh38p1.chrY (purple) and GRCh38p2.chrY (cyan) are completely overlapped by GRCh38p7.chrY (yellow), demonstrating they are identical. Compared to GRCh37p13.chrY (blue), the descendents GRCh38p1.chrY (purple), GRCh38p2.chrY (cyan) and GRCh38p7.chrY (yellow) have an extra turning-changed long sharp straight line (marked by a black arrow), respectively.

To create another alternative form (Fig. 3), we separately construct the 3D-trajectory map (X–Y–Z) and 2D-trajectory maps (X–Y, X–Z, Y–Z, X–Length, Y–Length, Z–Length) and combine them (Fig. 3). We calculate six genomes (in total 330.00 Mbp) of the human Y chromosomes (HuRef.chrY, YH.chrY, GRCh37p13.chrY, GRCh38p1.chrY, GRCh38p2.chrY and GRCh38p7.chrY) to create the three-dimensional coordinates (in total 9.03 GB), and thus create an alternative GGFM (Fig. 3). All tasks are completed in 12 hours on the TH-2 supercomputer.

The GGFMs (Figs 2 and 3) present the effective genomes since our *GenomeFingerprinter* algorithm^19^ only calculates non-N (A, T, G and C) bases, bypassing gaps and linking two non-N bases adjacent to the distal ends of a homopolymer of Ns. We deleted the homoploymers of Ns in total 34.19 Mbp to get the cleaned 25.65-Mbp sequence from 59.84-Mbp GRCh37p13.chrY. And we deleted the homoploymers of Ns in total 30.67 Mbp to get the cleaned 26.41-Mbp sequence from 51.08-Mbp GRCh38p1.chrY. One homopolymer of Ns (3.04 Mbp, from 10,248,904 bp to 13,291,760 bp) of GRCh37p13.chrY is cleaned. And 37 homopolymers of Ns (in total 1.43 Mbp, dispersed from 10,413,615 bp to 11,840,896 bp) of GRCh38p1.chrY are cleaned, but the remaining scattered non-N bases (in total 1.61 Mbp) are calculated, resulting in a “jumping” line (marked by a black arrow) on the GGFM (Fig. 3). These deleted Ns at specific positions are marked by a box with black dash lines on the GGFM (Fig. 3).

### Detecting discrepancies among the six assemblies

We compare the human Y chromosomes on the GGFM (Figs 2 and 3). HuRef.chrY is distinct due to its incomplete sequence at q-arm terminus. YH.chrY and GRCh37p13.chrY share an architecture, as confirmed by their similar 2D-contour maps (Fig. 2c,d). GRCh38.chrY has multiple releases (e.g., GRCh38p1.chrY, GRCh38p2.chrY and GRCh38p7.chrY), but they are factually identical (Fig. 2e,f). The 2D-contour maps indicate that GRCh37p13.chrY is distinct from GRCh38p1.chrY and GRCh38p2.chrY (Fig. 2d–f). There is an extra turning-changed long sharp straight line on the GGFM (Fig. 3), which can distinguish GRCh37p13.chrY from GRCh38p1.chrY (and throughout GRCh38p7.chrY). Hence we intuitively display and detect the incredible discrepancies of chromosome architecture among HuRef.chrY, YH.chrY, GRCh37p13.chrY, GRCh38p1.chrY, GRCh38p2.chrY and GRCh38p7.chrY (Fig. 3). These findings validate that GRCh.chrY and YH.chrY have a better quality of chromosome architecture over HuRef.chrY, and suggest that GRCh37p13.chrY or GRCh38p1.chrY is likely misassembled (Fig. 3).

### Deleting the misassembled chromosome architecture

**Figure 4:**
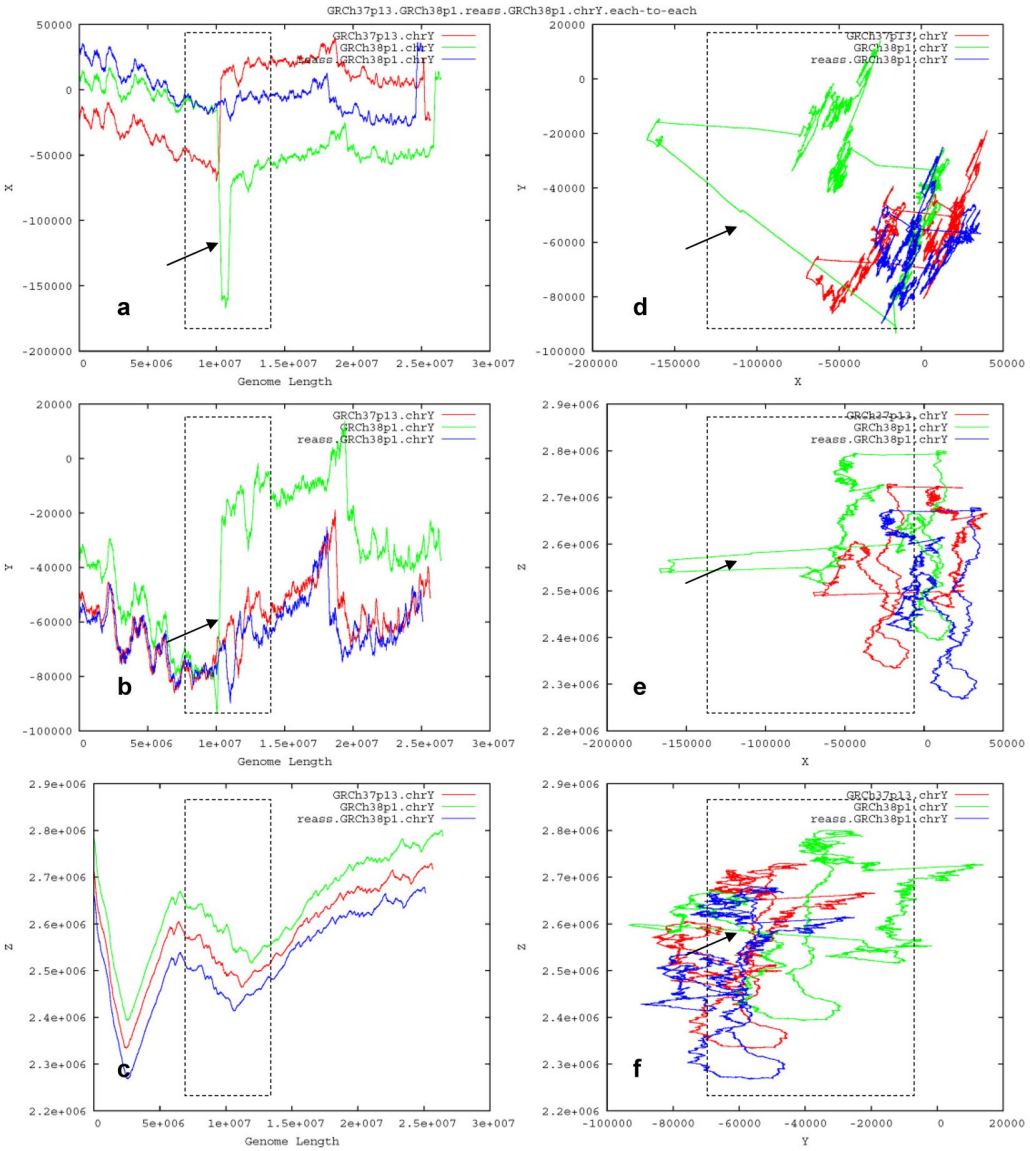
Deleting the misassembled segment of the human Y chromosome GRCh38p1.chrY. Compared to GRCh37p13.chrY (red), GRCh38p1.chrY (green) is likely misassembled, as identified by the turning-changed long sharp straight line (marked by a black arrow) on the GGFM. Guided by the long sharp straight line (**a**), the PRED-deletion of a 1.30-Mbp segment of GRCh38p1.chrY created the re-assembled form, reass.GRCh38p1.chrY (blue), which rescues the rough landscape compatibility of chromosome architecture and matches its antecedent GRCh37p13.chrY (red).

To evaluate which one is likely misassembled, we need to understand the mechanism of how the turning-changed long sharp straight line occurs between GRCh37p13.chrY and GRCh38p1.chrY (Fig. 3). The logic is simple with no biases: if the antecedent GRCh37p13.chrY were (and should have been over time) correct, then the descendent GRCh38p1.chrY with a newly-introduced extra segment should be incorrect; and vice versa. To simplify the logic of validations, here we deliberately hypothesise that the turning-changed long sharp straight line on the GGFM (Fig. 3) is resulted from likely misassembling of GRCh38p1.chrY. To test this, we delete the identified segment corresponding to the long sharp straight line on the GGFM (Fig. 3). Specifically, we delete a 1.30-Mbp (from 9,999,936 bp to 11,299,986 bp) target segment (Supplementary Dataset 1) from the prior-cleaned GRCh38p1.chrY, as guided by the turning-changed long sharp straight line on the 2D-trajectory map (X–Length) of the GGFM (Fig. 4a). As expected, the resulting re-assembled form (reass.GRCh38p1.chrY) of GRCh38p1.chrY does roughly match its antecedent GRCh37p13.chrY on the GGFM (Fig. 4). Hence, we name a proofreading errors-deletion (PRED) for this operation of target deletion guided by the GGFM (Fig. 4a). Such a PRED-deletion rescues the harmonious state of chromosome architecture among the relatives on the GGFM (Fig. 4). Accordingly, we name a landscape compatibility for such an observed harmonious state.

To justify such a rescue (Fig. 4), we decompose and analyse sub-constituents of the 1.30-Mbp target segment (Supplementary Dataset 1). Before that, we must exclude interferences by the telomeric and centromeric regions that were masked by Ns in GRCh38p1.chrY. We prior-deleted Ns before conducting the PRED-deletion, which ensures the deleted 1.30-Mbp target segment (Supplementary Dataset 1) is devoid of Ns. As anticipated, we scan it but find no tandem repeats (TTAGGG)n, regardless of dispersed 116 copies of single TTAGGG element. These data conclude the 1.30-Mbp segment (Supplementary Dataset 1) is not from a telomeric region featured by the known tandem repeats (TTAGGG)n^15^, thus leaving the centromeric region to be a suspect.

### Re-locating the deleted target segment

**Figure 5:**
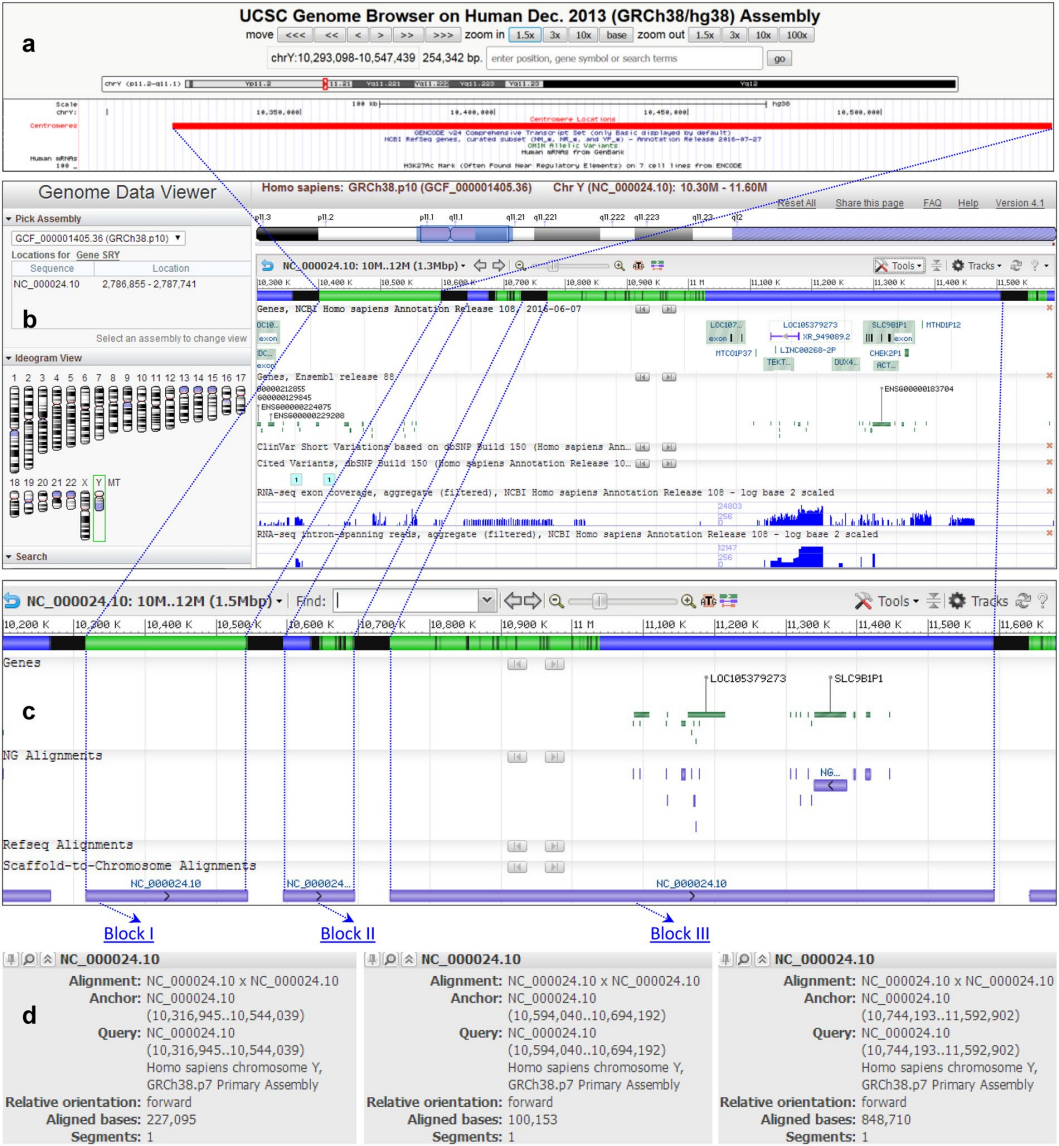
Re-locating the target segment. The 1.30-Mbp segment of GRCh38p1.chrY covers three blocks scattered in the modelled centromeric and pericentromeric region of GRCh38p10.chrY.

We re-locate the 1.30-Mbp target segment (Supplementary Dataset 1) against the newest assembly GRCh38p10 (GCF_000001405.36). The UCSC Human BLAT analysis^26^ indicates a match in centromeric region (Fig. 5a). The NCBI Genome Data Viewer (Fig. 5b) illustrates that it fits in a broad region of GRCh38p10.chrY with three blocks (Fig. 5c): Block I (227.10 Kbp, from 10,316,945 bp to 10,544,039 bp), Block II (100.15 Kbp, from 10,594,040 bp to 10,694,192 bp), and Block III (848.71 Kbp, from 10,744,193 bp to 11,592,902 bp) (Fig. 5d). Such three blocks constitute the assigned centromeric and pericentromeric region (1.18 Mbp, from 10,316,945 bp to 11,592,902 bp) of GRCh38p10.chrY (Fig. 5), which roughly equals the assigned gap placeholder (3.04 Mbp, from 10,248,904 bp to 13,291,760 bp) of GRCh37p13.chrY.

To justify our findings (Fig. 5), we survey the literatures but find no documents describing how such a megabase-sized target segment was assembled step by step. We track out that Block I (227.10 Kbp) was documented to be the DYZ3 alpha satellite array in a centromeric database that was created from the HuRef WGS reads library^16^, whereas Block II (100.15 Kbp) and Block III (848.71 Kbp) are unclear (Fig. 5) about their assembling processes that caused dramatic changes from GRCh37p13.chrY to GRCh38p1.chrY (and throughout GRCh38p7) (Figs 3 and 4). We conclude that the 1.30-Mbp target segment (Supplementary Dataset 1) does locate in the centromeric and pericentromeric region of GRCh38p1.chrY (and throughout GRCh38p10.chrY) (Figs 3, 4 and 5), which encourages us to trace its sub-constituents that contributed to the observed turning-changed long sharp straight line on the GGFM (Figs 3 and 4).

### Tracing BACs of the deleted target segment

**Figure 6:**
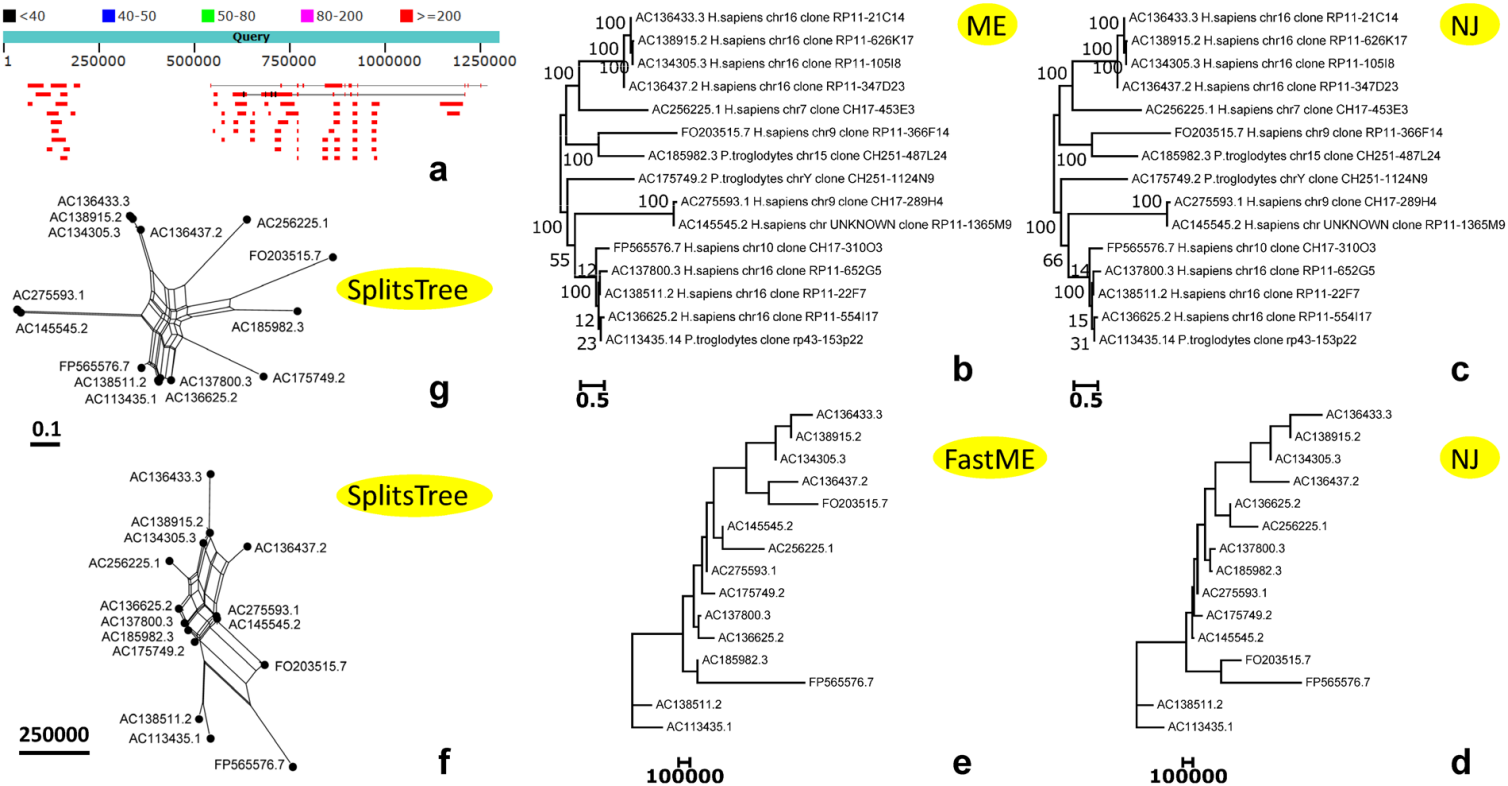
Tracing BACs in the target segment. BLAST search with the 1.30-Mbp segment against the NCBI nr/nt database hits divergent homologous BACs (**a**). The chosen 15 BACs are displayed on the un-rooted phylogenetic trees (**b**,**c**,**d**,**e**) and phylogenetic networks (**f**,**g**). The bootstrap consensus ME (**b**) and NJ (**c**) trees constructed by the MEGA6 package have a low confidence at arguable sub-branches. But the NJ (**d**) and FastME (**e**) trees constructed by our method have a better resolution at the questionable sub-branches. The SplitsTree networks (**f**,**g**) constructed by using our weighted distance matrix (**f**) and the MEGA distance matrix (**g**), respectively, illustrate that our method has a better resolution for the taxa containing high copy numbers of repeats (e.g., we track out that FP565576.7 has a 35.74-Kbp segment of (TGGAA)_7149_ in the main text).

BLAST search against NCBI nr/nt database with the 1.30-Mbp segment (Supplementary Dataset 1) shows no hits over the entire megabase-sized sequence, but traces homologous BACs (>150.0 Kbp) (Fig. 6a). With cover >20% and identity >80%, we choose 15 BACs to compose a dataset for phylogenetics analysis (Fig. 6b–g). The results demonstrate that the traced 15 BACs are divergent homologues stemmed from the human (*H. sapiens*) autosomal chr16, chr10, chr9 and chr7 as well as from the chimpanzee (*P. troglodytes*) autosomal chr15 and sex chrY (Fig. 6b, c), and imply that these BACs might be shared or contaminated. Given that the 1.30-Mbp target segment (Supplementary Dataset 1) presents debuting in GRCh38p1.chrY (rather than in GRCh37p13.chrY) (Figs 3 and 4), but absents from HuRef.chrY and YH.chrY that did not use BACs for sequencing and assembling, they are unlikely shared.

Note that our method is 2,880 times faster to create a distance matrix for such chosen 15 BACs. Our method took only 1 minute to calculate genome fingerprints and create a weighted distance matrix, but the MEGA6 package^22^ took 48 hours to complete base-to-base alignments and calculate a pair-wise distance matrix (i.e., the MEGA distance matrix). We use the MEGA6 package^22^ to construct traditional bootstrap consensus trees, both the ME (minimum-evolution) tree (Fig. 6b) and the NJ (neighbour-joining) tree (Fig. 6c) have a low confidence at arguable sub-branches (e.g., containing FP565576.7, AC_138511.2, AC_113435.1, AC_137800.3 and AC_136625.2). In contrast, we use our weighted distance matrix to construct an NJ tree (Fig. 6d) by NEIGHOR.exe (from the Phylip package)^23^ and a FastME tree (Fig. 6e) by FastMe software^21^ (an update version of ME). Our trees (Fig. 6 d,e) demonstrate a better resolution at the questionable sub-branches observed on the opposite MEGA trees (Fig. 6b,c). Further, we construct two phylogenetic networks (Fig. 6f,g) using SplitsTree4 software^24,25^ based on our weighted distance matrix and the MEGA distance matrix, respectively. They are approximate to one another, but ours (Fig. 6f) has a better resolution for discriminating the major discrepancies (e.g., FP565576.7, AC185982.3 and FO203515.7) observed on the phylogentic trees (Fig. 6b–e). Accordingly, we track out that FP565576.7 (114.29 Kbp, *H. sapiens* chr10 clone CH17-310O3) has a 35.74-Kbp segment of 7,149 copies of a 5-bp (TGGAA) unit (i.e., a cluster of (TGGAA)_7149_); AC185982.3 (179.50 Kbp, *P. troglodytes* chr15 clone CH251-487L24) has a 53.18-Kbp segment of 311 copies of a 171-bp unit; and FO203515.7 (147.99 Kbp, *H. sapiens* chr9 clone RP11-366F14) has a 9.00-Kbp segment of 1,801 copies of a 5-bp (TCATT) unit. These findings demonstrate that our method has a better resolution for taxa containing high copy numbers of repeats. We thus use our method to construct phylogenetic networks throughout the next sections when dealing with a number of large and divergent sequences, on which traditional approaches may not work.

### Tracing interspersed repeats of the deleted target segment

We search the 1.30-Mbp target segment (Supplementary Dataset 1) against the RepeatMasker/Repbase database^27^ and summarise about 2,720 hits of known repeats (Supplementary Table S1), including (1) 1,156 interspersed repeats (in total 350.57 Kbp) such as DNA transposons, LTR retrotransposons, and non-LTR retrotransposons; (2) 1,396 tandem repeats (in total 584.95 Kbp) such as satellite DNA; and (3) 168 endogenous retrovirus (in total 58.17 Kbp). These data highlight the likelihood that such large-scale repeats (up to 76.43% of the 1.30-Mbp segment) are responsible for its likely misassembling. Given that most known repeats are short (Supplementary Table S1), preventing us from exhaustively analysing them one by one, we intend to evaluate the chosen examples of predicted long repeats (e.g., LTR retrotransposons and satellite DNA). Hence we conduct *de novo* predictions of long repeats from the 1.30-Mbp segment (Supplementary Dataset 1), trace homologues, and analyse evolutionary relationships.

**Figure 7:**
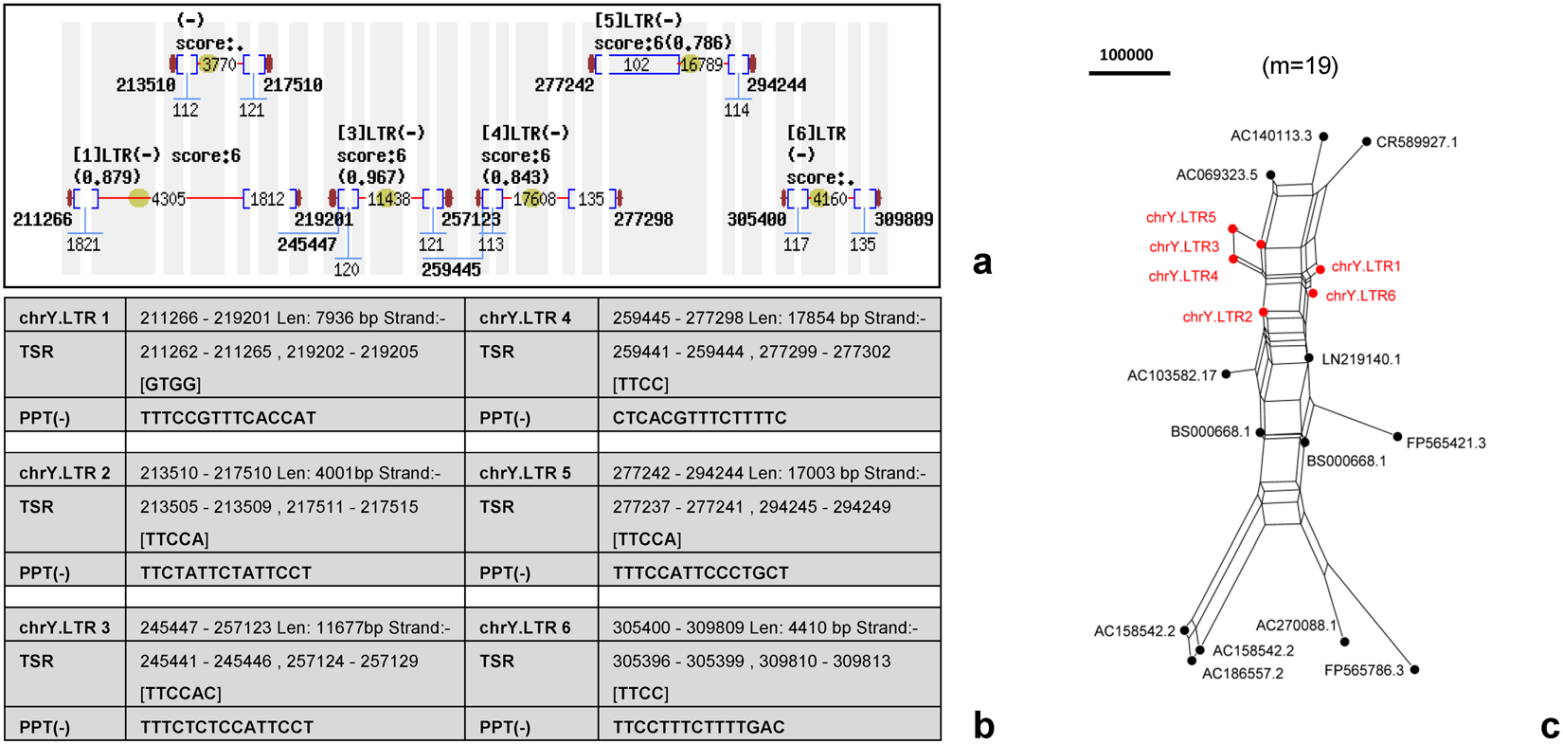
Tracing LTR retrotransposons in the target segment. Six LTR retrotransposons scattered on a 98.54-Kbp cluster (**a**) with key features (**b**) are predicted from the 1.30-Mbp target segment. These 6 LTR retrotransposons (red) are mono-centred on the phylogenetic network (**c**).

Using LTR-FINDER software^28^, we predict 6 LTR retrotransposons (Fig. 7a,b) that are dispersed on a 98.54-Kbp cluster (from 211,266 bp to 309,809 bp) of the 1.30-Mbp segment (Supplementary Dataset 1). We use each of them to do the BLAST search against the NCBI nr/nt database and select top 10 hits (if applicable) to compose a dataset for phylogenetics analysis. Such LTR retrotransposons are mono-centred on the phylogenetic network (Fig. 7c), coinciding with their close locations (Fig. 7a,b). These findings weaken the impacts of interspersed repeats, thus strengthen the impacts of tandem repeats to be elucidated.

### Tracing tandem repeats of the deleted target segment

**Figure 8:**
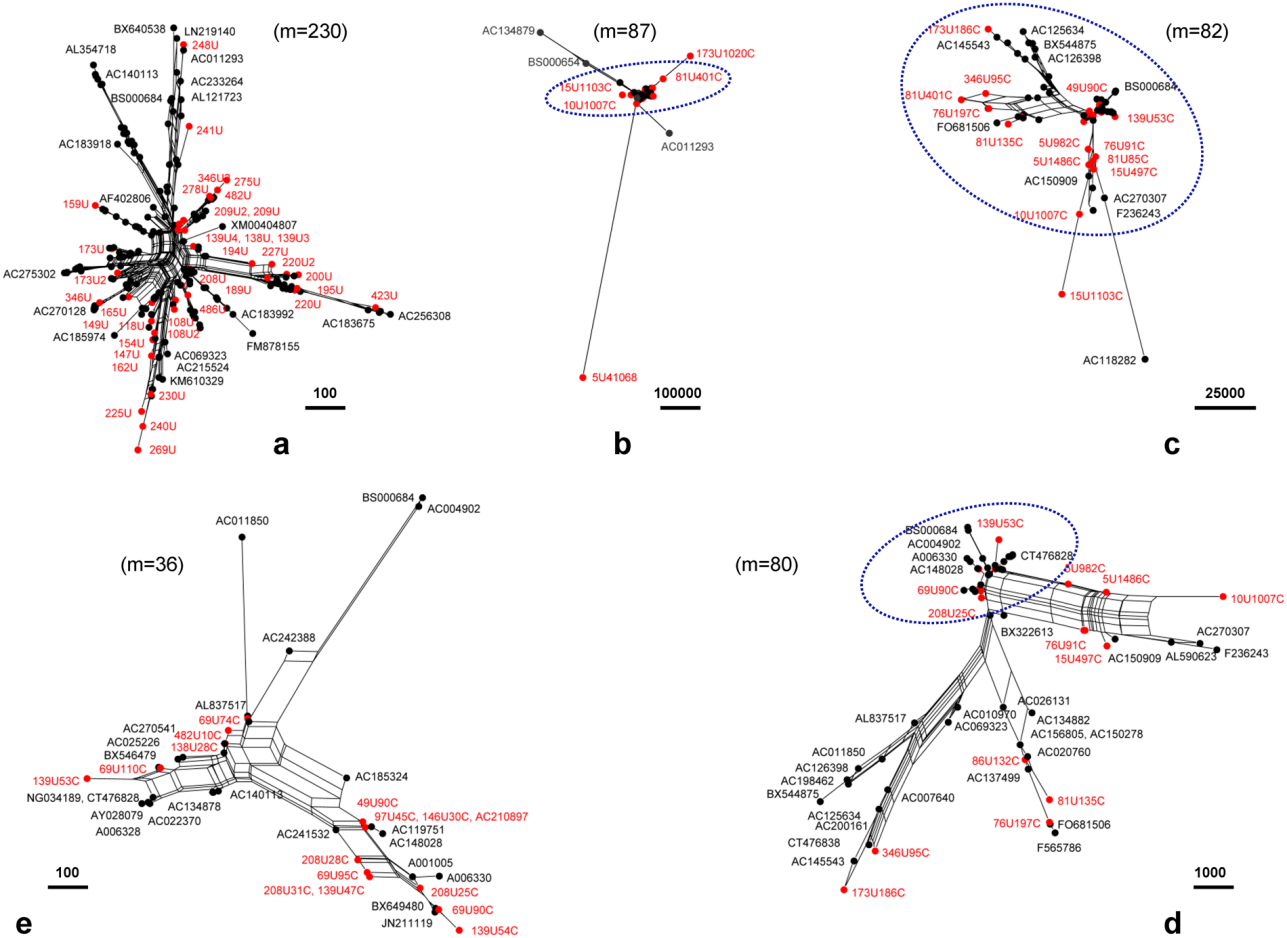
Tracing tandem repeats in the target segment. The traced (black) homologues (with GenBank IDs) and the predicted (red) core-units (CUs) (**a**) and core-unit repeats (CURs) (**b**,**c**,**d**,**e**) from the 1.30-Mbp segment randomly distribute on phylogenetic networks (**a**,**b**,**c**,**d**,**e**), indicating an observed individual landscape compatibility. But the orphan CURs (**b**) such as 5U41068C (205.34 Kbp) and 173U1020C (176.46 Kbp) are exceptional outliers, suggesting distribution biases. The insets (**c**,**d**,**e**) from the centre of (**b**) are enlarged in a cascade manner (m is the number of sequences therein), with representatives labelled for clarity.

Using TRF software^29^, we predict 2,531 tandem repeats (Supplementary Dataset 2), more than the records (1,396 tandem repeats) in the RepeatMask/Repbase database^28^ (Supplementary Table S1). The TRF program^29^ can list “period size” and “consensus size”, which are usually the same. We use the former for simple notation throughout this paper. We name core-units (CUs) (e.g., 173U for a 173-bp monomer) and core-unit repeats (CURs) (e.g., 173U1020C for 1,020 copies of 173U). A CUR is composed of multiple copies of a CU. The core-unit repeats (CURs) here are equivalent to the higher-order repeats (HOR) elsewhere^16^. For instance, 173U1020C containing 1,020 copies of 173U (a 173-bp monomer) yields a 176.46-Kbp segment (from 7 to 175,880 bp) of the 1.30-Mbp segment, which corresponds to Block I (227.10 Kbp) in the assigned centromeric region of GRCh38p10.chrY (Fig. 5). Likewise, 5U41068C containing 41,068 copies of 5U (a 5-bp monomer, GAATG) yields a 205.34-Kbp segment (from 995,979 to 1,203,802 bp) of the 1.30-Mbp segment, which corresponds to a part of Block III (848.71 Kbp) in the assigned pericentromeric region of GRCh38p10.chrY (Fig. 5). Each is close to a BAC’s size (>150.0 Kbp). We choose long tandem repeats to do the BLAST search against the NCBI nr/nt database, and select top 10 hits (if applicable) from each search to compose a dataset for pursuing our phylogenetics analysis. We construct phylogenetic networks both at the CUs level (Fig. 8a) and at the CURs level (Fig. 8b–e). Under the circumstances tested, all CUs (Fig. 8a) and most CURs (Fig. 8c–e) with their traceable homologues demonstrate random distributions, respectively, in a harmonious state (i.e., a landscape compatibility), regardless of certain outliers (Fig. 8b).

Such random distributions on the phylogenetic networks, both at the CUs level (Fig. 8a) and at the CURs level (Fig. 8c–e), demonstrate individual landscape compatibility among relatives. But the orphan BAC-sized CURs (e.g., 5U41068C and 173U1020C) indicate distribution biases to be exceptional outliers on the phylogenetic network (Fig. 8b), betraying such an observed landscape compatibility. Hence we group the CURs into two categories. Category 1 contains most CURs that randomly distribute on the phylogenetic networks (Fig. 8c–e), obeying the observed landscape compatibility. Category 2 contains the orphan CURs that distribute as exceptional outliers on the phylogenetic network (Fig. 8b), betraying the observed landscape compatibility. These orphan CURs include 173U1020C (176.46 Kbp, from 7 to 175,880 bp), 15U1103C (16.54 Kbp, from 448,092 to 464,937 bp), 5U41068C (205.34 Kbp, from 995,979 to 1,203,802 bp), and 81U401C (32.48 Kbp, from 1,171,087 to 1,203,802 bp). These data conclude that all CUs (Fig. 8a) and most CURs (Fig. 8c–e) are of reasonable quality, having traceable homologues; but the orphan BAC-sized CURs (Fig. 8b) are likely over-repeated (up to 33.14% of the 1.30-Mbp segment, see Supplementary Dataset 1 and Dataset 2), lacking traceable homologues at this moment.

## Discussion

This paper presents a novel geometric analysis method (called *GenomeLandscaper*) (Fig. 1) to conduct landscape analysis of genome-fingerprints maps (GFM) in order to trace large-scale repetitive regions and retrospectively assess their impacts on global architectures of assembled chromosomes (Figs 2, 3, 4, 5, 6, 7 and 8). We develop an alignment-free and bootstrap-free method for phylogenetics analysis. This study also sets up an itinerary of using the *GenomeLandscaper* method (Figs 3, 4, 5, 6, 7 and 8). As a proof-of-concept study, we created a galaxy of genome-fingerprints maps (GGFM) (Figs 2, 3 and 4) for the human Y chromosomes (GRCh.chrY^12,13^, HuRef.chrY^4–6^ and YH.chrY^7^) and conducted multifaceted assessments on their global architectures (Figs 2, 3, 4, 5, 6, 7 and 8). Through data-mining approach without prior knowledge or biases (Fig. 1), our data-driven computational analyses (Figs 3, 4, 5, 6, 7 and 8) uncovered and characterised the questionable 1.30-Mbp target segment (Supplementary Dataset 1) that distinguished GRCh38p1.chrY (and throughout GRCh38p10.chrY) from GRCh37p13.chrY, HuRef.chrY and YH.chrY (Figs 2, 3, 4 and 5). We elucidated that (1) it was the 1.30-Mbp target segment (Supplementary Dataset 1), identified in the modelled centromeric and pericentromeric region debuting in GRCh38p1.chrY throughout GRCh38p10.chrY (Fig. 5), that contributed to the observed long sharp straight line on the GGFM (Figs 3 and 4); and (2) the orphan BAC-sized CURs (Fig. 8b) such as 173U1020C (176.46 Kbp) and 5U41068C (205.34 Kbp) were the major components (up to 33.14%) of the 1.30-Mbp segment (Supplementary Dataset 1 and Dataset 2). This proof-of-concept study validates the efficacy of our *GenomeLandscaper* method (Fig. 1). Hence we have established an effective method to display, detect, delete and analyse the large-scale repetitive regions, thus retrospectively assessing their impacts on the global architectures of assembled chromosomes. We expect that the *GenomeLandscaper* method could be broadly applicable to understanding and assessing the yet-untouchable centromeric and pericentromeric regions of variants from the human 1,000 genomes project^2,3^ and other mammalian genomes. Such endeavours should benefit improvements of the reference genome GRCh38^1^ and the 1,000 genomes^2,3^, which is valuable for precise medicine since the roles of centromeres in chromosomal behaviours and clinical diseases are increasingly appreciated^30,31^.

We would emphasise the technical features of our *GenomeLandscaper* method (Fig. 1). First, our *GenomeFingerprinter* algorithm circularises a linear sequence (Fig. 1a) to avoid interference from arbitrary cut-off sites of a sequence^19^, thus ensures the circularised 3D-map (Figs 1d and 2a) solely representing the given sequence. This feature improves the accuracy of computing a distance matrix (Fig. 1g) based on values of geometric centres of the circularised 3D-maps (Figs 1d and 2a). Second, we operate map-to-map comparison (Figs 2, 3 and 4), instead of base-to-base comparison (see Fig. 5), to relieve computation burdens. This alignment-free method allows us comparing large genomes in a big-picture view at a large scale (Figs 2, 3 and 4). Third, we weight the geometrical mean of radiuses (equation (2)) of the circularised 3D-maps to create a weighted distance matrix (Fig. 1g), thus discriminate a pair of overlapped 3D-maps that share a geometric centre but have different shapes. This feature improves the accuracy of clustering both distant and close relatives (Figs 6, 7 and 8). Fourth, we create a weighted distance matrix (Fig. 1g) using the values of geometric centres of the circularised 3D-maps (Fig. 1d), and conduct calculations in the mathematical real number system (equations (1) to (6)). Whenever calculating the same set of sequences should result in the same weighted distance matrix (Fig. 1g), thus leading to the sole phylogenetic tree (Fig. 1h) and phylogenetic network (Fig. 1i). As exemplified by the small-sized mitochondria genomes (<17.0 Kbp) of the primates, our tree was approximate to the traditional bootstrap consensus tree (Fig. 1). So did the moderate-sized BAC clones (>150.0 Kbp) (Fig. 6). Our NJ and FastME trees (Fig. 6d,e) and SplitsTree network (Fig. 6f) have a better resolution for the taxa containing high copy numbers of repeats. Our alignment-free and bootstrap-free method is faster and has worked effectively for cluster approximations in our cases (Figs 6, 7 and 8). Altogether, these features allow us analysing a number of large genomes (Mbp) (Figs 2, 3 and 4) and divergent sequences (variations in size, gap, and divergence) (Figs 6, 7 and 8), on which the traditional approaches^21–25^ may not work.

We have demonstrated main findings with significance throughout the proof-of-concept study. We have found that there is a landscape compatibility of chromosome architecture among relatives under comparison (Figs 3 and 4). A misassembled segment can be detected if and only if it breaks such a landscape compatibility (Fig. 3), which can guide the PRED-deletion of the misassembled segment (Fig. 4). As such, we have traced down the questionable 1.30-Mbp target segment (Supplementary Dataset 1) from GRCh38p1.chrY throughout GRCh38p10.chrY (Figs 3, 4 and 5). Furthermore, we have found multiple lines of evidence on its likely misassembling of the 1.30-Mbp target segment (Supplementary Dataset 1). First, we located it in the modelled centromeric and pericentromeric region of GRCh38p10.chrY (Fig. 5). Second, we decomposed it into sub-constituents such as BACs (Fig. 6), interspersed repeats (Fig. 7) and tandem repeats (Fig. 8). And we traced back their homologues from heterogeneous chromosomes beyond the human Y chromosome (Figs 6, 7 and 8). Third, we elucidated that among the examined tandem repeats, all CUs (Fig. 8a) and most CURs (Fig. 8c–e) were of reasonable quality, but the orphan BAC-sized CURs (up to 33.14% of the 1.30-Mbp segment) were likely over-repeated (Fig. 8b). Such data-driven analyses without prior knowledge or biases (Fig. 1) should offer an informative starting-point for the community to retrospectively verify the modelled centromeric and pericentromeric regions that caused dramatic changes from GRCh37p13.chrY to GRCh38p1.chY (and throughout GRCh38p10.chrY) (Figs 3, 4, 5 and 8). This proof-of-concept study demonstrates the power of our *GenomeLandscaper* method (Fig. 1), as discussed below.

The mode of map-to-map (instead of base-to-base, see Fig. 5) comparison (Figs 2, 3 and 4) not only overcomes computational constraints at a large scale, but also performs a holistic comparison in a big-picture view, thus bypassing the lack of a “true” sequence being referred to. Further, our *GenomeFingerprinter* algorithm^19^ only calculates non-N (A, T, G and C) bases, bypassing gaps and linking two non-N bases adjacent to the distal ends of a homopolymer of Ns, thus presents the effective genome. These advantages allow us to assess incomplete genomes regardless of gaps, sizes and divergences. A batch of incomplete genomes can be displayed as a GGFM (Figs 2, 3 and 4) and compared at the chromosome architecture level. As a result, we have detected the incredible discrepancies among the human Y chromosomes (GRCh.chrY^12,13^, HuRef.chrY^4–6^ and YH.chrY^7^), as well as the four releases (GRCh37p13.chrY, GRCh38p1.chrY, GRCh38p2.chrY and GRCh38p7.chrY) of the GRCh.chrY assembly *per se* (Figs 2, 3 and 4).

There is an observed individual landscape compatibility among relatives on the GGFM (Fig. 4) and the phylogenetic network (Fig. 8a,c–e), respectively. This feature enables us to display and detect a misassembled chromosome architecture in a big-picture view at a large scale (Fig. 3). Further, an individual trajectory map on the GGFM (Figs 3 and 4) exhibits its own sensitivity, an extent of disturbing the landscape compatibility among relatives. The 3D-trajectory map (X–Y–Z) (Fig. 2a) and three 2D-trajectory maps (X–Y, X–Z, Y–Z) (Figs 3d–f and 4d–f) are the most sensitive, another two 2D-trajectory maps (X–Length, Y–Length) (Figs 3a,b and 4a,b) are less sensitive, and the 2D-trajectory map (Z–Length) (Figs 3c and 4c) is the least sensitive. Hence, the components *Xn* and *Yn* of the three-dimensional coordinates carry more information about the distribution biases of bases. The 3D-trajectory map (X–Y–Z) (Figs. 2a) and 2D-trajectory map (X–Y) (Figs 3d and 4d) amplify such effects of both *Xn* and *Yn* on disturbing the landscape compatibility, thus yielding more complex and sensitive profiles. Two 2D-trajectory maps (X–Length, Y–Length) (Figs 3a,b and 4a,b) are easier (due to simplicity) to guide the PRED-deletion of an identified misassembled segment (Figs 3 and 4). In addition, the 2D-contour map (X–Y) (Fig. 2b–f) with high-resolution genome fingerprints is the most sensitive in discriminating close relatives (HuRef.chrY, YH.chrY, GRCh38p1.chrY and GRCh38p2.chrY).

The proofreading errors-deletion (i.e., the PRED-deletion) guided by a GGFM is an efficient means for deleting a misassembled segment (Fig. 4). We deliberately hypothesised that the extra turning-changed long sharp straight line on the GGFM (Fig. 3) resulted from likely misassembling of GRCh38p1.chrY, when compared to its antecedent GRCh37p13.chrY. This hypothesis simplified the logic of validations: if the antecedent were (and should have been over time) correct, then the descendent with a newly-introduced extra segment should be incorrect; and vice versa. Accordingly, we conducted the PRED-deletion of the 1.30-Mbp segment (Supplementary Dataset 1), guided by the turning-changed long sharp straight line on the GGFM (Fig. 4a). To justify its likely misassembling, we found multiple lines of evidence. First, the PRED-deletion of the 1.30-Mbp target segment (Supplementary Dataset 1) from GRCh38p1.chrY (Fig. 4) did rescue the landscape compatibility, matching its antecedent GRCh37p13.chrY. Second, its sub-constituents such as BACs (Fig. 6), LTR retrotransposons (Fig. 7), and tandem repeats (Fig. 8) had homologues traced from heterogeneous chromosomes beyond the human Y chromosome. Third, the orphan BAC-sized CURs such as 173U1020C (176.46 Kbp) and 5U41068C (205.34 Kbp) to be exceptional outliers on the phylogenetic network (Fig. 8b) were the major components (up to 33.14%) of the 1.30-Mbp segment (Supplementary Dataset 1 and Dataset 2), which mainly contributed to the turning-changed long sharp straight line on the GGFM (Figs 3 and 4).

Furthermore, to justify our findings from data-driven computational analyses, we conducted retrospective researches in literatures. We tracked out the centromere model representations debuting in GRCh38p1.chrY (DYZ3 0.23 Mbp) and GRCh38p1.chrX (DXZ1 3.60 Mbp), where the assigned gap placeholders were replaced by the models (DYZ3 and DXZ1)^16^ that were simulated based on a centromere database derived from the HuRef WGS reads library^6^. GRCh38p1.chrY bears a modelled centromere^1,16^, rather than a real one that was assembled from original reads. These facts are consistent with our findings. First, the 1.30-Mbp target segment (Supplementary Dataset 1) of GRCh38p1.chrY (from 9,999,936 bp to 11,299,986 bp) roughly equals the assigned gap placeholder of GRCh37p13.chrY (from 10,248,904 bp to 13,291,760 bp). Second, the 1.30-Mbp segment covers three blocks that are scattered on GRCh38p10.chrY (Fig. 5). Block I (227.10 Kbp) (Fig. 5) was documented to be the DYZ3 (0.23 Mbp) alpha satellite array^16^ that was simulated from the HuRef WGS reads library^6^. Block II (100.15 Kbp) and Block III (848.71 Kbp) (Fig. 5) remained unclear about their assembling processes that have caused dramatic changes from GRCh37p13.chrY to GRCh38p1.chrY throughout GRCh38p10.chrY (Figs 3, 4 and 5). Third, the orphan BAC-sized CURs (Fig. 8b) are the individual parts of Block I (227.10 Kbp) and Block III (848.71 Kbp), which locate in the centromeric and pericentromeric regions of GRCh38p10.chrY (Fig. 5). For instance, 173U1020C (176.46 Kbp, from 7 to 175,880 bp) locates on Block I (227.10 Kbp) in the centromeric region^16^. Likewise, 15U1103C (16.54 Kbp, from 448,092 to 464,937 bp), 5U41068C (205.34 Kbp, from 995,979 to 1,203,802 bp) and 81U401C (32.48 Kbp, from 1,171,087 to 1,203,802 bp) locate on Block III (848.71 Kbp) in the pericentromeric region (Figs 5 and 8b). Fourth, these orphan BAC-sized CURs are exceptional outliers on the phylogenetic network (Fig. 8b). They are scattered on and up to 33.14% of the 1.30-Mbp segment (Supplementary Dataset 1 and Dataset 2), which mainly contributed to the turning-changed long sharp straight line on the GGFM (Figs 3 and 4). Such findings coincide with the fact that the monomer ordering of the chromosome-specific alpha-satellite repeats was proportional to that observed in the initial read database, but the long-range ordering of repeats was inferred by a graph-based simulation in the modelled centromere of GRCh38p1.chrY^1,16–18^. We conclude that the orphan BAC-sized CURs (Fig. 8b) are likely over-repeated, lacking traceable homologues at this moment.

Meanwhile, the human satellites HSat2 and HSat3 were documented to be composed of 5-bp (e.g., CATTC, GAATG) repeats with diverged arrangements and constituted 1.5% of the human genome, occupying heterochromatic blocks adjacent to centromeric regions (see reference No.32 and others therein). By using the same graph-based simulation method^16^, the subfamily-specific 24-mers for HSat2 and HSat3 were recently modelled (each cluster is small, <20 Kbp) in the pericentromeric regions adjacent to centromeres of the human chromosomes 1 and Y^32^. The sizes of the predominant HSat3-rich arrays on the Y chromosomes were estimated to distribute differently within the distinct Y haplogroups^32^. The sizes of HSat3A6 (DYZ1) arrays from 396 individuals varied over an order of magnitude (7 to 98 Mbp)^32^. Thus we would suggest that both the core *k*-mers (i.e. CUs) and their copy numbers (i.e., CURs) should be equally concerned on the case-by-case basis. The modelled centromeric and pericentromeric regions debuting in GRCh38p1.chrY are not yet true, linear assemblies^1,16,32^; rather, they are modelled reference regions to be used for mimicking and mapping target short-reads at this stage. Hence the true CUs and CURs discussed in our cases remain to be elucidated in the future.

With and without the modelled centromeric and pericentromeric regions, the two counterparts (GRCh38p1.chrY and GRCh37p13.chrY) have provided excellent cases for a proof-of-concept study via data-mining without prior knowledge or biases, thus have validated the efficacy and demonstrated the power of our *GenomeLandscaper* method. The itinerary has worked effectively and should be generally applicable. However, we remind that it deserves to investigate whether the 1.30-Mbp segment (Supplementary Dataset 1) is a true intrinsic segment on the human Y chromosome. Theoretically, PCR reactions could be determinate, but could be undoable in this case owing to two-faceted difficulties: amplification of the 1.30-Mbp segment (Supplementary Dataset 1) as a whole is technically unachievable, while amplifying sizable parts of its elements is challenging in designing proper primers. The desired primers must cover the sub-constituents, such as BACs (Fig. 6), LTR retrotranspons (Fig. 7), and tandem repeats (Fig. 8), but also avoid similar sequences that might be scattered on the human Y chromosome. These tasks are hardly achievable due to the natures of eukaryotes repeats^27,33^. Historical experimental data (including physical mapping, see reference No.32 and others therein) would be inadequate at the level of single-base resolution to discriminate arguable sequences of repeats in genomics. We would recommend sequencing and assembling the real centromeric and pericentromeric regions of the human Y chromosomes by future technology (once it is applicable)^8,34–36^. With true, linear reference regions for pursuing our GGFM comparisons, one could ultimately justify whether the 1.30-Mbp segment (Supplementary Dataset 1) containing the orphan BAC-sized CURs (Fig. 8b) might result from an artefact or should be a true intrinsic segment. Therefore, the graph-based simulation method modelled the centromeric^16^ and pericentromeric^32^ regions in the human reference genome GRCh38^1^, which opened a door to model the yet-untouchable centromeric and pericentromeric regions of mammalian genomes. Our *GenomeLandscaper* method offers a geometric analysis means to retrospectively assess such modelled regions, which opens a window to assess their impacts on the global architectures of assembled chromosomes.

## Methods

### Calculation of three-dimensional coordinates and construction of a genome-fingerprints map

We calculated the three-dimensional coordinates for a genome using our *GenomeFingerprinter* algorithm^19^. We transformed a genome into a genome-fingerprints map (GFM); and a set of GFMs were simultaneously constructed to create a galaxy of genome-fingerprints maps (GGFM).

### Construction of phylogenetic tree and phylogenetic network

We calculated a weighted Euclidean distance matrix (as described in Results section). We used the weighted distance matrix to create a phylogenetic tree using FastME2.0 software^21^ under the minimum-evolution (ME) model, and using NEIGHBOR.exe (from the Phylip package 3.695)^23^ under the neighbour-joining (NJ) model. We used the weighted distance matrix to create a phylogenetic network using SplitsTree4.0 software^24,25^ under the neighbour-net model. Default parameters were applied. We used the MEGA6 package^22^ to conduct pair-wise and multiple alignments by Clustal W (with IUB DNA weight matrix, transition weight 0.5, gap opening penalty 15, gap extension penalty 6.66)^22^, and create a minimum-evolution (ME) tree and a neighbour-joining (NJ) tree (both with bootstrap replicates 100, maximum composite likelihood model, nucleotide substitutions, transition and transversion)^22^. The pair-wise deletion model was used for the treatment of gaps and missing subset data^22^.

### Retrieval of known repeats and prediction of interspersed repeats and tandem repeats

For a given sequence, we searched it against RepeatMasker/Repbase database (at http://www.girinst.org/censor/index.php) to retrieve the registered repeats^27^. We used LTR-FINDER software^28^ and TFR software^29^ to conduct *de novo* predictions from the given sequence in order to predict LTR retrotransposons and tandem repeats, respectively. The parameters for LTR-FINDER were default, while for TFR were (2/7/7/80/10/50/500).

### Data availability

YH.chrY was downloaded from http://yh.genomics.org.cn/. HuRef.chrY (AC_000156.1), GRCh37p13.chrY (NC_000024.9), GRCh38p1.chrY (NC_000024.10 as of December 15, 2013), GRCh38p2.chrY (NC_000024.10 as of March 25, 2015), GRCh38p7.chrY (NC_000024.10 as of June 10, 2016), and mitochondria genomes NC_012920.1 (*Homo sapiens*), NC_001643.1 (*Pan troglodytes*), NC_011120.1 (*Gorilla gorilla*), NC_012670.1 (*Macaca fascicularis*) and NC_005943.1 (*Macaca mulatta*) were downloaded from NCBI database. The Supplemental Information from this study was provided online.

## Electronic supplementary material

Supplementary Information

Dataset 1

Dataset 2
