# Supplementary material for: GenomeLandscaper: Landscape analysis of genome-fingerprints maps assessing chromosome architecture
Source: Sci Rep. 2018 Jan 18;8:1026. doi: 10.1038/s41598-018-19366-2 (PMC5773709; doi:10.1038/s41598-018-19366-2)
Supplement: Supplementary file 1 — Supplementary Information [file 41598_2018_19366_MOESM1_ESM.pdf]

GenomeLandscaper: Landscape analysis of genome–fingerprints maps assessing chromosome architecture

Hannan Ai[1,2], Yuncan Ai[1, *], Fanmei Meng[1]

[1] State Key Laboratory for Biocontrol, School of Life Sciences, Sun Yat–sen University, Guangzhou, Guangdong 510275, China

[2] Department of Electrical and Computer Engineering, College of Engineering, University of Illinois at Urbana–Champaign, Urbana, IL 61801, USA

[*]Corresponding Author

Supplementary Information

Table S1. Known repeats in RepeatMask/Repbase predicted from the 1.30-Mbp target segment

Dataset 1. PRED.cleaned_GRCh38p1.chrY.xlsx

Sequence of the 1.30-Mbp target segment deleted from the cleaned GRCh38p1.chrY.

Dataset 2. TRF.PRED.cleaned_GRCh38p1.chrY.xlsx

Tandem repeats predicted from the 1.30-Mbp target segment.

Table S1. Known repeats in RepeatMask/Repbase predicted from the 1.30-Mbp target segment

Repeat Class	Fragments	Length (bp)
**Interspersed Repeat**	**1156**	**350570**
**DNA transposon**	**415**	**123147**
Academ	1	36
Crypton	3	159
CryptonV	1	68
Dada	1	51
EnSpm/CACTA	62	16569
Ginger1	2	196
Ginger2/TDD	1	84
Harbinger	12	835
Helitron	19	16919
Kolobok	2	153
Mariner/Tc1	39	12510
Merlin	1	58
MuDR	197	82192
P	6	549
Polinton	7	511
Sola	7	563
Sola1	4	267
Sola2	3	296
Zisupton	1	82
Transib	1	65
hAT	37	4140
piggyBac	3	217
ERV1	54	17935
ERV2	9	6037
ERV3	91	30154
**LTR Retrotransposon**	**164**	**25361**
BEL	8	543
Copia	34	4905
DIRS	3	247
Gypsy	109	16361
**Non-LTR Retrotransposon**	**577**	**202062**
CR1	22	5018
CRE	2	198
Daphne	3	225
I	1	63
L1	213	114815
L2	4	458
Nimb	1	55
Penelope	79	25483
R1	2	105
R4	1	121
RTE	3	157
RTEX	2	232
SINE	238	54509
SINE1/7SL	216	51583
SINE2/tRNA	22	2926
Tx1	5	585
**Tandem Repeat**	**1396**	**584954**
Satellite	1396	584954
**Endogenous Retrovirus**	**168**	**58167**
**Total**	**2720**	**993691**
